# A Case of Local Spontaneous Combustion

**Published:** 1826-07

**Authors:** 


10. *Local Spontaneous Combustion.* We confess that we have always been sceptical as to the alledged phenomenon of spontaneous combustion. Nor does the following case diminish our scepticism. It is related in Hecker's Annals for 1825, volume the second. A young woman, while working at her needle, on the evening of January 21st, 1825, perceived a sudden and extraordinary heat pervade her whole body, and at the same time a violent burning sensation in the fore finger of her left hand, which became *encircled with a blue flame,* to the extent of an inch and a half, emitting a sulphureous smell. *Affusions of cold water and a wetted napkin had no effect in extinguishing the flame.* The whole hand was then immersed in water, but, like the famous Greek fire, nothing could put it out. The young woman ran home, in doing which the flame communicated with her clothes. When she got home she kept applying milk to the hand during the whole of the night, and at length succeeded in extinguishing this mysterious combustion. On the 25th, she was brought to the general hospital of Hamburgh, when the finger and wrist were found covered with small blisters, such as take place in burns or scalds. The patient had intense thirst, and head-ache, but no other febrile or morbid symptoms. We need not pursue the details of this case. She was not discharged till the 5th May, having, during the greater part of this long sojourn, been afflicted with burning sensation in the hand, and startings (tressaillemens) in different parts of the body. We cannot doubt the occurrences which took place in hospital; but, till the combustion-scene is authenticated by better testimony than this young woman's ipse dixit, we shall beg leave to withhold our belief in the truth of her assertions.

